# Architecture of Amylose Supramolecules in Form of Inclusion Complexes by Phosphorylase-Catalyzed Enzymatic Polymerization

**DOI:** 10.3390/biom3030369

**Published:** 2013-07-11

**Authors:** Jun-ichi Kadokawa

**Affiliations:** 1 Graduate School of Science and Engineering, Kagoshima University, 1-21-40 Korimoto, Kagoshima 890-0065, Japan; 2 Research Center for Environmentally Friendly Materials Engineering, Muroran Institute of Technology, 27-1 Mizumoto-cho, Muroran, Hokkaido 050-8585, Japan

**Keywords:** Amylose supramolecule, hydrophobic interaction, inclusion complex, vine-twining polymerization, selective inclusion

## Abstract

This paper reviews the architecture of amylose supramolecules in form of inclusion complexes with synthetic polymers by phosphorylase-catalyzed enzymatic polymerization. Amylose is known to be synthesized by enzymatic polymerization using α-d-glucose 1-phosphate as a monomer, by phosphorylase catalysis. When the phosphorylase-catalyzed enzymatic polymerization was conducted in the presence of various hydrophobic polymers, such as polyethers, polyesters, poly(ester-ether), and polycarbonates as a guest polymer, such inclusion supramolecules were formed by the hydrophobic interaction in the progress of polymerization. Because the representation of propagation in the polymerization is similar to the way that a vine of a plant grows, twining around a rod, this polymerization method for the formation of amylose-polymer inclusion complexes was proposed to be named “vine-twining polymerization”. To yield an inclusion complex from a strongly hydrophobic polyester, the parallel enzymatic polymerization system was extensively developed. The author found that amylose selectively included one side of the guest polymer from a mixture of two resemblant guest polymers, as well as a specific range in molecular weights of the guest polymers poly(tetrahydrofuran) (PTHF) in the vine-twining polymerization. Selective inclusion behavior of amylose toward stereoisomers of chiral polyesters, poly(lactide)s, also appeared in the vine-twining polymerization.

## 1. Introduction

Polysaccharides are naturally occurring carbohydrate polymers, where each monosaccharide residue is linked directly through a glycosidic linkage in the main-chain [1]. The glycosidic linkage is a type of covalent bond that joins a monosaccharide residue to another group, which is typically another saccharide residue. Natural polysaccharides are found in various sources such as plant, animal, seaweed, and microbial kingdoms, which have specific and very complicated structures owing, not only to a structural diversity of monosaccharide residues, but also to the differences in stereo- and regio-configurations of glycosidic linkages. The large diversity of polysaccharide structures contributes to serve as vital materials for a range of important* in vivo* functions in host organisms, e.g., providing an energy resource, acting as a structural material, and conferring specific biological properties, and a subtle change in the chemical structure has a profound effect on the properties and functions of the polysaccharides [2,3,4]. Therefore, the preparation of artificial polysaccharides has attracted increasing attention because of their potential applications as materials in the fields related to medicine, pharmaceutics, cosmetics, and food industries. Polysaccharides are theoretically produced by the repeated reactions for the formation of a glycosidic linkage, so-called glycosylation of a glycosyl acceptor with a glycosyl donor [5,6,7,8]. To develop a superior method for the synthesis of polysaccharides by such repeated glycosylations, the* in vitro* approach by enzymatic catalysis i.e., enzymatic polymerization, has been significantly investigated [9,10,11,12,13,14,15] as enzymes have remarkable catalytic advantages compared with other types of catalysts in terms of the stereo- and regioselectivities. The enzymatic polymerization, therefore, is a very powerful tool for the stereo- and regioselective construction of polysaccharides under mild conditions, where monomers can be employed in their unprotected forms, leading to the direct formation of the unprotected saccharide chains in aqueous media.

**Figure 1:**
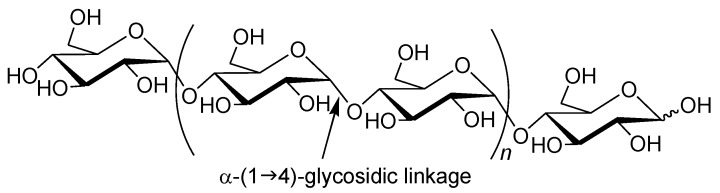
Structure of amylase.

**Figure 2:**
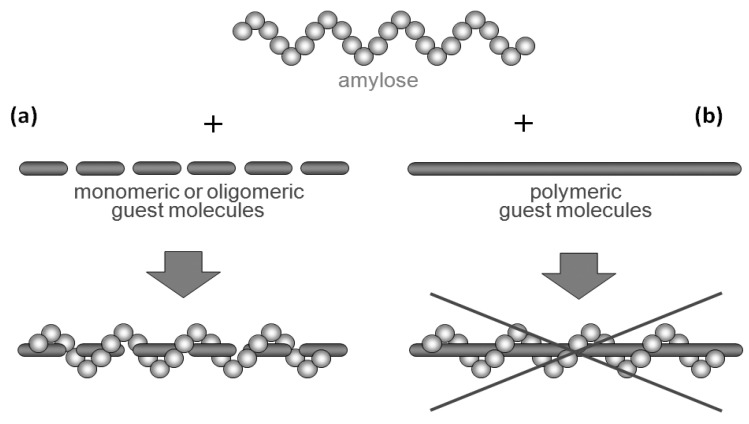
Amylose forms inclusion complex with relatively low molecular weight hydrophobic molecule (**a**); but, mostly, does not form it with polymeric molecule (**b**).

Amylose is a natural glucose polymer connected through α-(1→4)-glycosidic linkages (Figure 1) [1]. This is one component of starch and acts as an energy resource in nature with the other component of starch, that is, amylopectin, which has a branched structure composed of α-(1→4)-glucans with a small portion of α-(1→6)-glycosidic linkages [16]. Amylose has recently been recognized as a candidate as a high-performance polymeric material because it acts as a host molecule and forms polysaccharide supramolecules by inclusion complexation with various guest molecules of relatively low molecular weight (inclusion complexes) owing to the helical conformation (Figure 2a) [17]. The driving force for inclusion of guest molecules in the cavity is mainly host-guest hydrophobic interaction as the inside of the amylose helix has a hydrophobic nature due to the presence of hydrophilic hydroxy groups in the glucose residues on outer part of the helix. Therefore, hydrophobicity is generally in demand as the property of guest molecules to be included by amylose. Development of methods for the architecture of amylose supramolecules with polymeric guest molecules is a significant research topic to provide new self-assembled polymeric materials with regularly controlled nanostructures, which have potential to exhibit new high performance functions. However, only limited studies have been reported regarding the direct construction of inclusion complexes composed of amylose and polymeric molecules (Figure 2b) [18,19,20,21,22,23,24,25,26] as the driving force for the inclusion complexation of guest molecules into the cavity of amylose is the weak hydrophobic interaction as mentioned earlier, the amylose cavity does not have a sufficient ability to include the long chains of polymeric guests. The author has considered for the architecture of such amylose supramolecules, i.e., amylose-polymer inclusion complexes in the phosphorylase-catalyzed enzymatic polymerization field [14,15,27,28,29,30] as a structurally controlled amylose is efficiently synthesized by an enzymatic polymerization through phosphorylase catalysis [31,32,33,34,35,36,37]. Following the recent review article on the series of these studies [30], in this article, the author would like to deal with the comprehensive results and discussion of this approach, including the further progress of the investigation to precisely architect such amylose supramolecules in form of inclusion complexes between amylose and synthetic polymers by the phosphorylase-catalyzed enzymatic polymerization [38]. Specifically, the present review article is described on the basis of the viewpoint that the precision architecture of the regularly controlled polysaccharide supramolecules has been achieved by means of the enzymatic synthesis of structurally defined polysaccharides according to Section 2.

## 2. Characteristic Features of Phosphorylase-Catalyzed Enzymatic Polymerization to Produce Amylose

**Figure 3:**
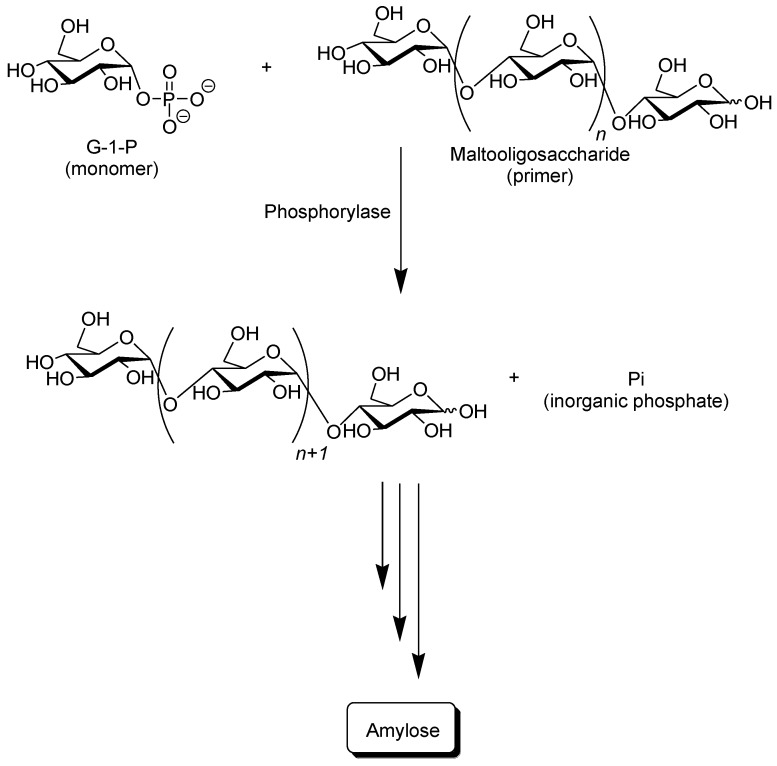
Phosphorylase-catalyzed enzymatic polymerization of G-1-P to form amylase.

Phosphorylase catalyzes the reversible phosphorolysis of α-(1→4)-glucans at the nonreducing end, such as glycogen and starch, in the presence of inorganic phosphate to produce α-d-glucose 1-phosphate (G-1-P) [31]. By means of the reversibility of the enzymatic reaction, α-(1→4)-glycosidic linkage can be constructed by the phosphorylase-catalyzed glycosylation using G-1-P as a glycosyl donor. As a glycosyl acceptor, maltooligosaccharides with degrees of polymerization DPs higher than the smallest one recognized by phosphorylase are used. The smallest glycosyl acceptor for the phosphorylase-catalyzed glycosylation is typically known to be maltotetraose (G_4_), whereas that for phosphorolysis is typically maltopentaose (G_5_). In the glycosylation, a glucose unit is transferred from G-1-P to a nonreducing end of the glycosyl acceptor to form α-(1→4)-glycosidic linkage. When the excess molar ratio of G-1-P to the glycosyl acceptor is present in the reaction system, the successive glycosylations, i.e., the enzymatic polymerization of G-1-P as a monomer, occurs to produce the α-(1→4)-glucan chain, that is, amylose (Figure 3) [32,33,34,35,36,37]. The polymerization is initiated from a nonreducing end of the glycosyl acceptor, and thus, it is often called a “primer.” Because the phosphorylase-catalyzed enzymatic polymerization proceeds analogously to a living polymerization, the polydispersity of the amylose produced is narrow (*M*_w_/*M*_n_ < 1.2) and its molecular weight can be controlled by the G-1-P/primer feed molar ratios. Phosphorylase is the only enzyme that can produce amylose with the desired average molecular weight [39]. 

**Figure 4:**
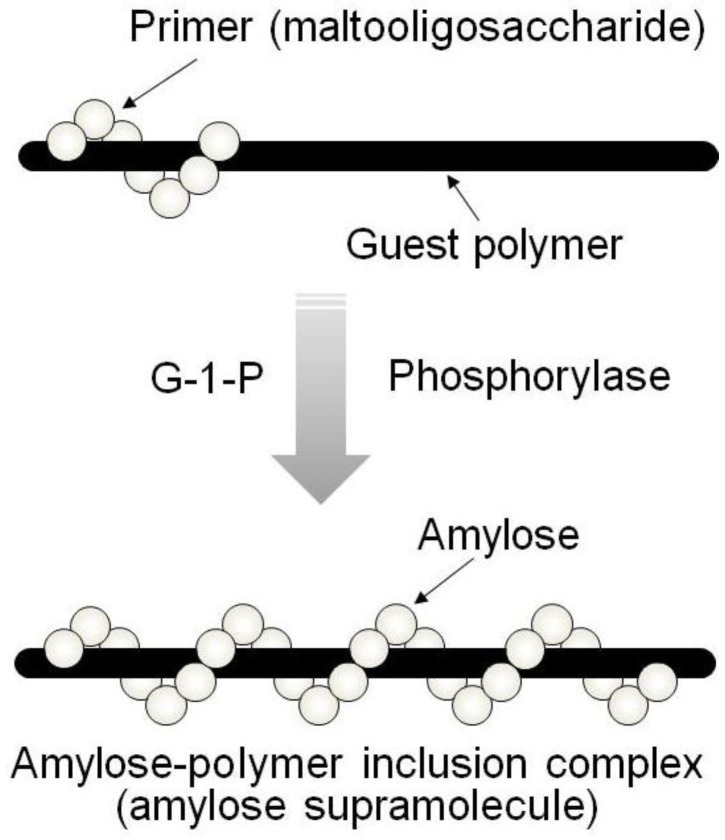
Image of “vine-twining polymerization”.

By means of the phosphorylase-catalyzed enzymatic polymerization for direct synthesis of amylose, the author has investigated developing an efficient method for the architecture of inclusion complexes of amylose with synthetic polymers. The representation of propagation in the polymerization system mirrors the way that the vine of a plant grows, twining around a rod. Accordingly, the author has proposed that this polymerization method for the architecture of amylose-polymer supramolecular inclusion complexes be named “vine-twining polymerization” (Figure 4) [14,15,27,28,29,30].

## 3. Architecture of Amylose-Poly(tetrahydrofuran) Inclusion Complex by Vine-Twining Polymerization

**Figure 5:**
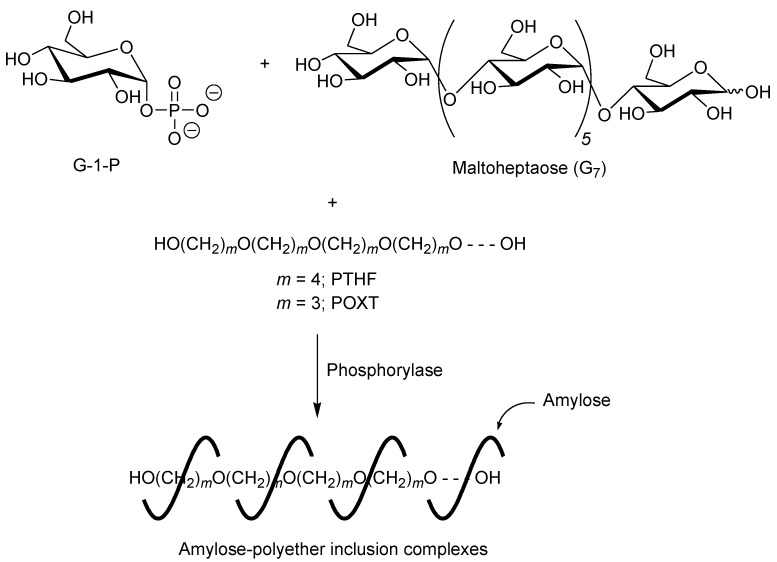
Architecture of inclusion complexes by vine-twining polymerization using hydrophobic guest polyethers.

A first example of vine-twining polymerization was reported in the system using poly(tetrahydrofuran) (PTHF) as a hydrophobic guest polyether (Figure 5) [40]. When the phosphorylase-catalyzed enzymatic polymerization of G-1-P from maltoheptaose (G_7_) as a primer was conducted in the presence of hydroxy-terminated telechelic PTHF, with *M*_n_ of 4000 in sodium citrate buffer, the product was gradually precipitated during the progress of the polymerization, which was isolated by filtration and characterized by ^1^H NMR and powder X-ray diffraction (XRD) measurements.

**Figure 6:**
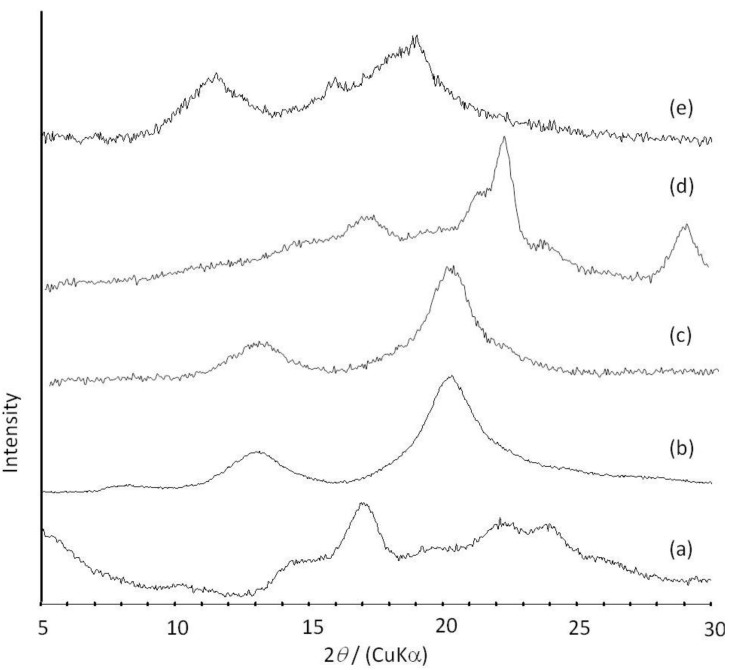
XRD profiles of amylose (**a**); amylose-PTHF inclusion complex (**b**); amylose-P(GA-*co*-CL) inclusion complex (**c**); the product obtained by vine-twining polymerization using P(GA-*b*-CL) (**d)**; and amylose-PLLA inclusion complex (**e**).

In the ^1^H NMR spectrum of the product in DMSO-*d*_6_, signals not only due to amylose but also due to PTHF were detected. Moreover, the methylene peak of PTHF was broadened and shifted to a higher magnetic field compared with that of a sole PTHF, suggesting that each methylene group in PTHF is immobile and interacts with the protons inside the amylose cavity by complexation. In addition, the NMR pattern assignable to amylose and PTHF was also observed by the measurement in NaOD/D_2_O solvent. A sole PTHF was not dissolved with NaOD/D_2_O, and thus, no peak due to PTHF appeared in the ^1^H NMR spectrum of a mixture of PTHF with NaOD/D_2_O. These NMR results indicated that PTHF in the product was solubilized in alkaline solution, probably by suppressing the formation of crystalline aggregates because of its inclusion complexation in the cavity of amylose. The XRD profile of the product showed two strong peaks at 2*θ* = 12.4 and 19.8° (Figure 6b), which was completely different from that of a sole amylose (Figure 6a), but similar to that of inclusion complexes of amylose with monomeric guest molecules reported in a previous study [41]. The above NMR and XRD results strongly supported that the amylose-PTHF inclusion complex was obtained by the vine-twining polymerization system.

The formation of an inclusion complex was not observed by mixing amylose and PTHF in a buffer solvent, strongly suggesting its formation during the progress of the enzymatic polymerization in the above system. To additionally study the relation between the formation of an inclusion complex and the enzymatic polymerization process, the following experiment was conducted. When PTHF was added to the reaction solution immediately after the enzymatic polymerization of G-1-P had initiated, the identical inclusion complex to the aforementioned system was produced. However, the contents of PTHF in the products decreased as the time between the initiation of the enzymatic polymerization and the addition of PTHF to the solution increased. These results revealed that amylose did not sufficiently include PTHF in the cavity after the polymerization produced amylose with relatively higher molecular weight. Accordingly, it was considered that the inclusion complex had only been formed simultaneously with the progress of the enzymatic polymerization according to the “vine-twining” process.

The effect of molecular weights of PTHFs on the formation of inclusion complexes in the vine-twining polymerization was investigated by using those with various molecular weights (1000, 2000, 10,000, and 14,000) [42]. When PTHF with *M*_n_ of 1000 or 2000 was employed as the guest polymer, inclusion complexes were obtained in the vine-twining polymerization. In contrast, the use of PTHFs with higher *M*_n_s such as 10,000 and 14,000, in the vine-twining polymerization, did not induce the formation of inclusion complexes with amylose. The PTHFs with higher *M*_n_s were not sufficiently dispersed in buffer of the polymerization solvent, resulting in difficulty of the inclusion by amylose. To yield inclusion complexes from these PTHFs, the diethyl ether/buffer two-phase system was attempted for the vine-twining polymerization. The higher molecular weight PTHFs were first dissolved in diethyl ether, and then, buffer solvent was added to the ether solution (diethyl ether:buffer = 1:5 (v/v)). Then, the phosphorylase-catalyzed enzymatic polymerization of G-1-P was carried out with vigorously stirring the two-phase mixture. Consequently, the XRD profiles of the products from the higher molecular weight PTHFs obtained by the two-phase system indicated the formation of the inclusion complexes.

## 4. Architecture of Inclusion Complexes by Vine-Twining Polymerization Using Other Polyethers as Guest Polymers

To investigate the effect of alkyl chain lengths in polyethers on the formation of inclusion complexes in the vine-twining polymerization, the phosphorylase-catalyzed enzymatic polymerization of G-1-P was performed in the presence of polyethers with different alkyl chain lengths from PTHF (the number of methylenes = 4), that is, poly(oxetane) (POXT, the number of methylenes = 3) and poly(ethylene glycol) (PEG, the number of methylenes = 2) [42]. The ^1^H NMR spectrum of the product from POXT in DMSO-*d*_6_ showed the signals due to both amylose and POXT and its XRD pattern was same as that of the aforementioned amylose-PTHF inclusion complex. These analytical data indicated that the inclusion complex was formed by the vine-twining polymerization using POXT as the guest polyether (Figure 5). In the ^1^H NMR spectrum of the product from PEG, on the other hand, only the signals due to amylose were detected. Moreover, the XRD profile of the product from PEG showed the pattern for amylose, but did not exhibit that for an inclusion complex. These data indicated that amylose was produced by the enzymatic polymerization in this system, but that did not induce inclusion complexation with PEG. The above results were reasonably explained by the different hydrophobicities in the polyethers. The hydrophilic nature of PEG caused much less hydrophobic interaction with the cavity of amylase, resulting in no complexation by amylose, whereas the hydrophobic polyethers such as PTHF and POXT interacted with the cavity of amylose, leading to the formation of the inclusion complexes by the vine-twining polymerization. The above results suggested that the hydrophobicity of guest polymers strongly affected whether inclusion complexation takes place in the vine-twining polymerization system.

## 5. Architecture of Inclusion Complexes by Vine-Twining Polymerization Using Carbonyl-Containing Hydrophobic Polymers as Guest Polymers

**Figure 7:**
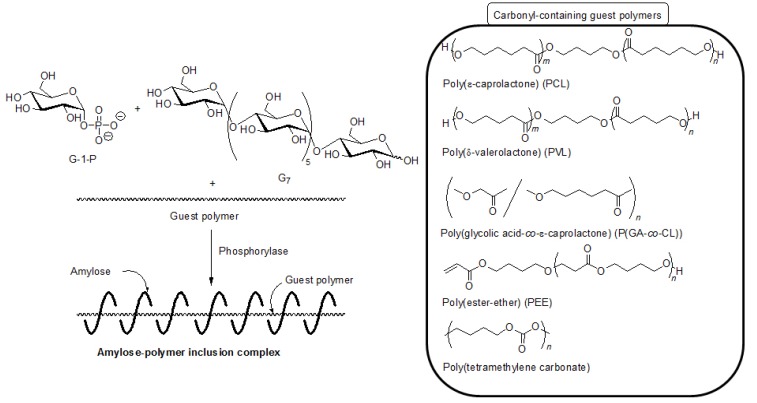
Architecture of inclusion complexes by vine-twining polymerization using carbonyl-containing hydrophobic polymers.

**Figure 8:**
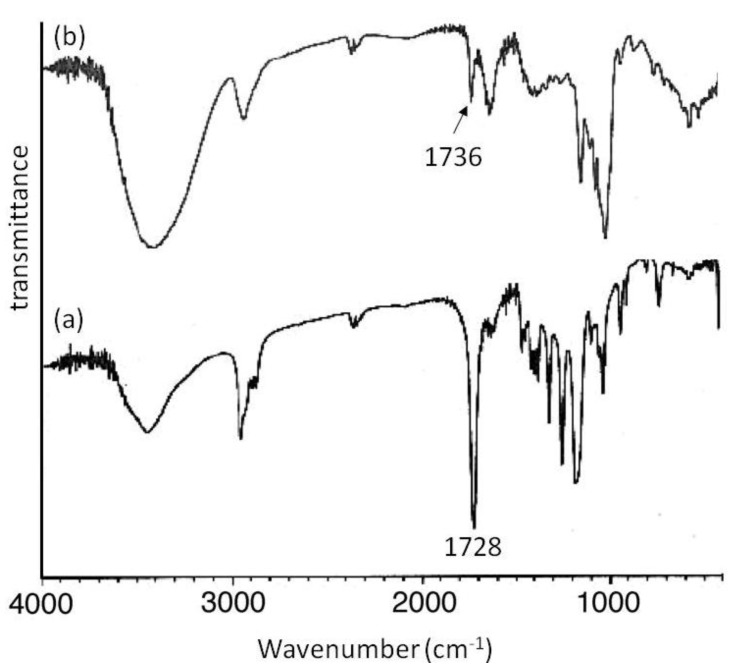
IR spectra of PVL (**a**); and amylose-PVL inclusion complex (**b**).

Based on significance in the hydrophobicity of guest polymers on the formation of inclusion complexes, well-known hydrophobic polyesters, that is, hydroxy-terminated telechelic poly(ε-caprolactone) (PCL) and poly(δ-valerolactone) (PVL) were employed as the guest polymer in the vine-twining polymerization (Figure 7) [43,44]. The phosphorylase-catalyzed enzymatic polymerization of G-1-P from G_7_ was conducted in the presence of PCL or PVL in sodium citrate buffer and the precipitated products were characterized by ^1^H NMR and XRD measurements. The ^1^H NMR spectra of the products from PCL with *M*_n_ of 1000 and PVL with *M*_n_ of 2000 showed signals not only due to amylose but also due to the polyesters. The XRD patterns of the products were completely different from those of a sole amylose and were similar as those of the aforementioned amylose-PTHF inclusion complex. Moreover, the IR spectrum of the original PVL exhibited a strong absorption at 1728 cm^−1^ (Figure 8a), corresponding to a carbonyl group of the crystalline PVL, and which of PVL in the product shifted to the region at 1736 cm^−1^ (Figure 8b) assignable to the non-crystalline PVL. This result suggested that the crystalline PVL did not present in the product because of inclusion of a PVL chain in the cavity of amylose, suppressing the formation of crystalline aggregates among PVL chains. All the above analytical results supported the structures of the inclusion complexes of amylose with PCL and PVL.

Although PCL with a higher *M*_n_ (2000) was employed as the guest polyester in the vine-twining polymerization, it was not sufficiently dispersed in sodium citrate buffer of the polymerization solvent, and accordingly, the inclusion complex was not produced. To yield the inclusion complex from such a PCL, the vine-twining polymerization was attempted in a mixed solvent of acetone and sodium citrate buffer (1:5 (v/v)) as PCL with the *M*_n_ was dispersed in the mixed solvent system. The resulting product was characterized by the ^1^H NMR and XRD measurements to be the inclusion complex. As previously mentioned, the inclusion complex was formed from PVL with *M*_n_ of 2000 by the vine-twining polymerization in sodium citrate buffer, suggesting that PVL was more favorable as the guest polymer to form inclusion complex, compared with PCL by the vine-twining polymerization.

It was also found that amylose-poly(glycolic acid-*co*-ε-caprolactone) (P(GA-*co*-CL)) inclusion complexes were obtained by the vine-twining polymerization, using biodegradable P(GA-*co*-CL)s (Figure 7) [38]. As poly(glycolic acid) (PGA) shows high crystallinity and low dispersibility in aqueous buffer, resulting in the difficulty of the inclusion of PGA in the cavity of amylose, the copolyester, P(GA-*co*-CL) composed of the two biodegradable chains, was used. Thus, the vine-twining polymerization was carried out by the phosphorylase-catalyzed polymerization in the presence of P(GA-*co*-CL)s with various unit ratios in sodium acetate buffer. The ^1^H NMR spectra of the products showed the signals due to amylose and P(GA-*co*-CL) and their XRD patterns showed the typical diffraction peaks, assignable to inclusion complexes (Figure 6c). These results indicated that the products were the inclusion complexes composed of amylose and P(GA-*co*-CL). The ratios of GA unit to CL unit in P(GA-*co*-CL)s did not affect inclusion complexation by amylose. On the other hand, an inclusion complex was not formed in the vine-twining polymerization using poly(glycolic acid-*block*-ε-caprolactone) (P(GA-*b*-CL)), as confirmed by the XRD profile of the product (Figure 6d). As P(GA-*co*-CL) and P(GA-*b*-CL) exhibit amorphous and crystalline natures, respectively, it has been considered that the crystallinity of the guest copolyesters affected inclusion complexation by amylose. In addition, lipase-catalyzed hydrolysis of P(GA-*co*-CL), in the inclusion complex, was partly inhibited, likely because amylose surrounded P(GA-*co*-CL) to prevent the approach of lipase.

As the other guest polyester, a hydrophobic poly(ester-ether) (–CH_2_CH_2_C(=O)OCH_2_CH_2_CH_2_CH_2_O–), which was composed of alternating ester and ether bonds, was employed for the vine-twining polymerization (Figure 7) [44]. The structure of the product was evaluated by the ^1^H NMR and XRD measurements to be the inclusion complex. When a hydrophilic poly(ester-ether) (–CH_2_CH_2_C(=O)OCH_2_CH_2_O–), which had a shorter methylene length, was employed as the guest polymer, no inclusion complex was produced. This result also supported that the hydrophobicity of the guest polymers affected the formation of inclusion complexes by the vine-twining polymerization.

In addition, to no production of an inclusion complex from such a hydrophilic polymer, the formation of inclusion complexes, had not been achieved from polymers with strong hydrophobicity due to their aggregation in aqueous buffer solvent in the vine-twining polymerization, as an example using PCL with the higher *M*_n_ [44]. As the other example, the vine-twining polymerization using poly(oxepane), which was a more hydrophobic polyether owing to longer alkyl chains compared with PTHF and POXT, did not induce inclusion complexation by amylose [42].

**Figure 9:**
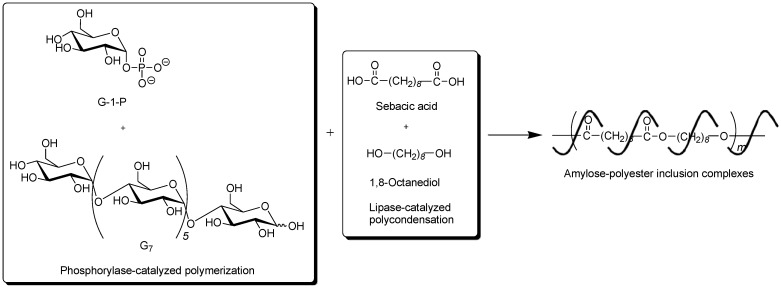
Architecture of inclusion complexes composed of amylose and strongly hydrophobic polyester in parallel enzymatic polymerization system.

To obtain the inclusion complex from a strongly hydrophobic polyester, the parallel enzymatic polymerization system, as an extensive approach of the vine-twining polymerization, was attempted [45]. In this system, two enzymatic polymerizations, which were the phosphorylase-catalyzed enzymatic polymerization of G-1-P from G_7_, producing amylose and the lipase-catalyzed polycondensation of a dicarboxylic acid and a diol, giving an aliphatic polyester of the guest polymer [46,47], were simultaneously performed (Figure 9). As the monomers for the polycondensation, sebacic acid and 1,8-octanediol were used, which were converted to the strongly hydrophobic polyester by the lipase-catalyzed polycondensation under aqueous conditions. The product by the parallel enzymatic polymerization system was evaluated by the ^1^H NMR and XRD measurements, which indicated the formation of the inclusion complex of amylose with the polyester. To confirm the fact that the inclusion complex was obtained only by the parallel enzymatic polymerization system, the following two experiments were conducted. When the phosphorylase-catalyzed enzymatic polymerization was carried out in the presence of such a strongly hydrophobic polyester, according to the vine-twining polymerization manner, amylose was produced, but it did not induce inclusion complexation with the polyester. As the other experiment, the lipase-catalyzed polycondensation of sebacic acid and 1,8-octanediol was carried out in the presence of amylose. Consequently, amylose included some monomers, but did not include the polyester although the enzymatic polycondensation progressed. These results concluded that the inclusion complex of amylose with such a strongly hydrophobic polyester was obtained only by the parallel enzymatic polymerization system.

Hydrophobic aliphatic polycarbonates were also used as the guest polymer in the vine-twining polymerization to prepare the corresponding inclusion complexes with amylose [48]. When the vine-twining polymerization was conducted, using poly(tetramethylene carbonate) as the guest polycarbonate in an acetone/aqueous buffer mixed solvent, the precipitated product was evaluated by ^1^H NMR, XRD, and IR measurements to be the amylose-poly(tetramethylene carbonate) inclusion complex (Figure 7). The effect of the methylene chain lengths in the polycarbonates on inclusion complexation in the vine-twining polymerization was further examined by using poly(tetramethylene carbonate), poly(octamethylene carbonate), poly(decamethylene carbonate), and poly(dodecamethylene carbonate) as a guest polycarbonate. Compared with the polycarbonate with the shorter methylene chain length, such as the above mentioned poly(tetramethylene carbonate), only lesser amounts of the polycarbonates, having longer methylene chain lengths, were included in the cavity of amylose. Such strongly hydrophobic polycarbonates were not sufficiently dispersed in the acetone/aqueous buffer mixed solvent, resulting in difficulty in the inclusion complexation by amylose.

## 6. Selective Inclusion Complexation by Amylose in Vine-Twining Polymerization

**Figure 10:**
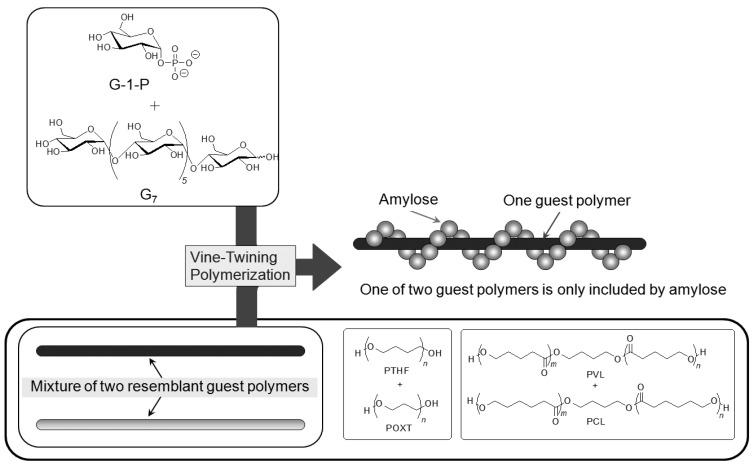
Amylose selectively includes one of two resemblant polyethers or polyesters.

On the basis of the aforementioned results, during the course of studies on the vine-twining polymerization, the author has considered that moderate hydrophobicity of guest polymers is in demand in deciding whether amylose includes them or not. Taking more precise information into account, amylose exhibits different inclusion behaviors depending on subtle changes in the structures of hydrophobic guest polymers. Such behavior of amylose was applied in the investigation to realize the selective inclusion complexation toward two resemblant guest polymers (Figure 10) [49,50]. For example, the selective inclusion of amylose was achieved by the vine-twining polymerization in the presence of a mixture of POXT (*M*_n_ = 1800) and PTHF (*M*_n_ = 1600) (unit molar ratio = 0.90:1.00) in sodium acetate buffer [49]. In the ^1^H NMR spectrum of the product (DMSO-*d*_6_), the signals due to PTHF and amylose were prominently detected, but the signals due to POXT, mostly, were not observed (POXT/PTHF = 0.02:1.00). This NMR result suggested that amylose almost selectively included PTHF from the mixture of the two resemblant polyethers in the vine-twining polymerization. The difference in the inclusion behavior of amylose toward the two polyethers was probably owing to the slight difference in their hydrophobicities. An attempt in the other selective inclusion system by amylose was made by the vine-twining polymerization in the presence of a mixture of two resemblant polyesters, that is, PVL (*M*_n_ = 830) and PCL (*M*_n_ = 930) (unit molar ratio = 1.00:0.92) [50]. In the ^1^H NMR spectrum of the product (DMSO-*d*_6_), the signals due to PVL and amylose were detected, whereas no signals due to PCL appeared, indicating that amylose selectively included PVL from the mixture of the two resemblant polyesters in the vine-twining polymerization.

The selective inclusion complexation toward a specific range in molecular weights of synthetic guest polymers by amylose in the vine-twining polymerization was also investigated [51]. As synthetic polymers have molecular weight distribution, they are reasonably considered as mixtures of analogous molecules with different numbers of the repeating units. Moreover, the number of repeating units contributes to the exhibition of different properties of the polymers with the same repeating unit. For example, PTHF with considerably low molecular weight is soluble in water, whereas that with higher molecular weight is insoluble in water, suggesting that the molecular weight of PTHF affects its hydrophobicity. Therefore, the vine-twining polymerization in the presence of three PTHFs with different *M*_n_s and (*M*_w_/*M*_n_)s (PTHF/1000; *M*_n_ and *M*_w_/*M*_n_ = 1350 and 2.86, PTHF/3000; *M*_n_ and *M*_w_/*M*_n_ = 3040 and 3.13, and PTHF/6000; *M*_n_ and *M*_w_/*M*_n_ = 6330 and 2.45) was investigated. When PTHF/3000 with *M*_w_/*M*_n_ = 3.13 was used as the guest polymer for the vine-twining polymerization, the *M*_w_/*M*_n_ of PTHF included by amylose became narrower (*M*_w_/*M*_n_ = 1.46) although its *M*_n_ (=3590) was comparable to that of the employed one (*M*_n_ = 3040). These results indicated that amylose selectively included a specific range in molecular weights of PTHF/3000 in the vine-twining polymerization. When the vine-twining polymerization using PTHF/1000, and PTHF/6000 as the guest polymer, was conducted, the *M*_n_s and (*M*_w_/*M*_n_)s of PTHFs included by amylose were similar as those using PTHF/3000 as aforementioned (the included PTHF/1000; *M*_n_ and *M*_w_/*M*_n_ = 3120 and 1.41, the included PTHF/6000; *M*_n_ and *M*_w_/*M*_n_ = 3700 and 1.74). Thus, it was concluded that amylose selectively included a specific range in molecular weights of PTHFs probably by the specific hydrophobic interaction.

**Figure 11:**
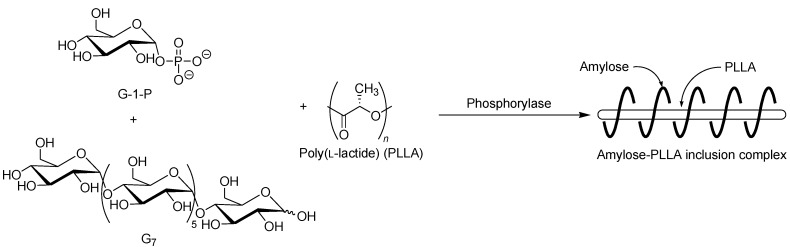
Stereoselective inclusion complexation by amylose in vine-twining polymerization using poly(l-lactide) (PLLA).

The selective inclusion complexation by amylose was also achieved in the vine-twining polymerization using chiral polyesters, i.e., poly(lactide)s (PLAs) as the guest polymer (Figure 11) [52]; there are three kinds of the stereoisomers, i.e., poly(l-lactide) (PLLA), poly(d-lactide) (PDLA), and racemic poly(dl-lactide) (PLDLA). When the vine-twining polymerization, using PLLA, was carried out, signals, not only due to amylose, but also due to PLLA, were observed in the ^1^H NMR spectrum of the product in DMSO-*d*_6_. The XRD pattern of the product showed two diffraction peaks at 2*θ* = 11°–12° and 18°–19° (Figure 6e), which was completely different from that of a sole amylose (Figure 6a), supporting the inclusion complex structure of the product. Interestingly, the diffraction peaks of the product were detected at lower angles compared with those of amylase-PTHF inclusion complex (Figure 6b) [40]. This difference in the two XRD patterns reasonably suggested that the diameter of amylose helix in the product was larger by inclusion of the bulky PLLA in the cavity of amylose. These analytical results fully supported that the amylose-PLLA inclusion complex was obtained in the vine-twining polymerization using PLLA as the guest polyester. To investigate the effect of the chirality in PLAs on inclusion by amylose, the vine-twining polymerization was performed using PDLA and PDLLA. Consequently, the ^1^H NMR spectra and the XRD patterns of the products indicated no formation of inclusion complexes. From the above results, it was concluded that amylose perfectly recognized the chirality in PLAs on inclusion complexation in the vine-twining polymerization.

## 7. Conclusions

In this article, the author reviewed the precision architecture of amylose supramolecules in the form of inclusion complexes by the phosphorylase-catalyzed enzymatic polymerization of G-1-P in the presence of hydrophobic synthetic polymers, such as polyethers, polyesters, a poly(ester-ether), and polycarbonates, according to the vine-twining polymerization manner. The corresponding amylose supramolecular inclusion complexes were formed in the process of the polymerization. The results in the vine-twining polymerization suggested that amylose exhibited different inclusion behaviors by the specific interactions with the guest polymers, depending on subtle changes in their structures. As of the production of the structurally defined polysaccharides by the enzymatic catalysis, the precision architecture of the regularly controlled amylose supramolecules, described herein, has been achieved. Moreover, the studies on the enzymatic architecture of the polysaccharide supramolecules have been based on the viewpoints that the greener and eco-friendly processes should be developed in the fields, not only of fundamental research, but also of practical application of the polymer and material chemistries. The polysaccharide supramolecules have increasingly been attracting a great deal of attention because of their potential for application as new functional materials in many research fields, such as medicine and pharmaceutics. Therefore, the present vine-twining polymerization method will be applied to the additional architecture of various amylose supramolecules with regularly controlled nanostructures, and accordingly contributes to providing new functional bio-based materials in the future [53,54].
